# Supplementary material for: Enhancing breast ultrasound segmentation through fine-tuning and optimization techniques: Sharp attention UNet
Source: PLoS One. 2023 Dec 13;18(12):e0289195. doi: 10.1371/journal.pone.0289195 (PMC10718429; doi:10.1371/journal.pone.0289195)
Supplement: S1 Appendix — (DOCX) [file pone.0289195.s001.docx]

Appendix A :
**A.1 Dice coefficient**

Dice coefficient is a measure of the similarity between two sets. It is defined as eq.9.

**A.2 Accuracy**

Accuracy is a measure of the overall performance of a binary classification model and is calculated as the ratio of correctly predicted pixels to the total number of pixels (eq.15).

| $\mathrm{Accuracy}= \frac{(TP + TN)}{(TP + FP + TN + FN)}$ | (A_1) |
| --- | --- |

where True Positive (TP) is the number of pixels that are correctly classified as positive, True Negative (TN) is the number of samples that are correctly classified as negative, False Positive (FP) is the number of samples that are incorrectly classified as positive, and False Negative (FN) is the number of samples that are incorrectly classified as negative.

**A.3 Precision**

Precision is a measure of the proportion of positive predictions that are correct and is calculated as the ratio of TP to the total number of predicted positives of each pixel.

| $\mathrm{Precision}= \frac{TP}{(TP + FP)}$ | (A_2) |
| --- | --- |

**A.4 Sensitivity**

Sensitivity, also known as recall or True Positive Rate (TPR), is a measure of the proportion of actual positive pixels that are correctly identified as positive by the model and is calculated as the ratio of TP to the total number of actual positives of each pixel.

| $\mathrm{Sensitivity}= \frac{TP}{(TP + FN)}$ | (A_3) |
| --- | --- |

**A.5 Specificity**

Specificity is a measure of the proportion of actual negative pixels that are correctly identified as negative by the model and is calculated as the ratio of TN to the total number of actual negatives of each pixel.

| $\mathrm{Specificity}= \frac{TN}{(TN + FP)}$ | (A_4) |
| --- | --- |

**A.6 F1 Score**

The F1 score is a measure of the harmonic mean of precision and sensitivity and provides a balanced evaluation of the model performance.

| $F1= \frac{2*(\mathrm{Precision}*\mathrm{Sensitivity})}{(\mathrm{Precision} + S\mathrm{ensitivity})}$ | (A_5) |
| --- | --- |

**A.7 Jaccard index**

The Jaccard Index, also known as Intersection over Union (IoU), is a measure used to quantify the similarity between two sets.

| $Jaccard index= \frac{Size of Intersection}{Size of Union}$ | (A_6) |
| --- | --- |
